# Jian-Pi-Gu-Shen-Hua-Yu Decoction Alleviated Diabetic Nephropathy in Mice through Reducing Ferroptosis

**DOI:** 10.1155/2024/9990304

**Published:** 2024-03-16

**Authors:** Shuquan Lv, Lirong Fan, Xiaoting Chen, Xiuhai Su, Li Dong, Qinghai Wang, Yuansong Wang, Hui Zhang, Huantian Cui, Shufang Zhang, Lixin Wang

**Affiliations:** ^1^Cangzhou Hospital of Integrated Traditional Chinese Medicine and Western Medicine of Hebei, Cangzhou 061012, Hebei, China; ^2^Botou Hospital of Traditional Chinese Medicine, Botou 062154, Hebei, China; ^3^Yunnan University of Chinese Medicine, Kunming 650500, Yunnan, China

## Abstract

**Background:**

Diabetic nephropathy (DN), one of the most frequent complications of diabetes mellitus, is a leading cause of end-stage renal disease. However, the current treatment methods still cannot effectively halt the progression of DN. Jian-Pi-Gu-Shen-Hua-Yu (JPGS) decoction can be used for the treatment of chronic kidney diseases such as DN, but the specific mechanism of action has not been fully elucidated yet.

**Purpose:**

The aim of this study is to clarify whether JPGS alleviates the progression of diabetic nephropathy by inhibiting ferroptosis.

**Materials and Methods:**

We established a DN mouse model to investigate the therapeutic effect of JPGS in a DN mouse model. Subsequently, we examined the effects of JPGS on ferroptosis- and glutathione peroxidase 4 (GPX4) pathway-related indices. Finally, we validated whether JPGS inhibited ferroptosis in DN mice via the GPX4 pathway using GPX4 inhibitor and ferroptosis inhibitors.

**Results:**

The results indicate that JPGS has a therapeutic effect on DN mice by improving kidney function and reducing inflammation. Additionally, JPGS treatment decreased iron overload and oxidative stress levels while upregulating the expression of GPX4 pathway-related proteins. Moreover, JPGS demonstrated a similar therapeutic effect as Fer-1 in the context of DN treatment, and RSL3 was able to counteract the therapeutic effect of JPGS and antiferroptotic effect.

**Conclusion:**

JPGS has significant therapeutic and anti-inflammatory effects on DN mice, and its mechanism is mainly achieved by upregulating the expression of GPX4 pathway-related proteins, thereby alleviating iron overload and ultimately reducing ferroptosis.

## 1. Introduction

Diabetic nephropathy (DN), a prevalent complication of diabetes mellitus (DM), stands as a prominent contributor to end-stage renal disease. In 2019, there were 460 million DM patients worldwide, of whom 30%–40% developed DN [1]. The pathogenesis of DN primarily involves inflammation, oxidative stress, renal hemodynamic changes, and dysregulated metabolism [2–5]. Medications that control blood glucose and lipids levels as well as blood pressure cannot impede the progression of DN [6]. Therefore, developing novel drugs to treat DN contributes significantly to prevention.

Ferroptosis, characterized by increased iron-dependent production of reactive oxygen species (ROS) and lipid peroxide, is a newly discovered form of regulated cell death [7]. Regulated by multiple genes with a complex mechanism involving iron homeostasis, lipid peroxidation, and metabolism, ferroptosis plays a pivotal role in the progression of renal diseases [8]. Ferroptosis contributes to DN through triggering renal tubular injury [9], glomerular injury [10], and renal fibrosis [11, 12]. A significant characteristic of DN is renal tubular injury, which emerges due to hyperglycemia-induced iron overload, diminished antioxidant, heightened ROS production, and escalated lipid peroxidation within renal tubular cells [13]. A novel approach to investigating the progression and treatment of DN may involve inhibiting ferroptosis, as indicated by recent studies [14, 15]. Thus, inhibiting ferroptosis in a targeted manner is expected to be an emerging strategy for improving DN-related renal injury.

Traditional Chinese medicine is efficacious in improving renal injury. Tang-Shen-Wei-Ning formula reduced podocyte injury in DN mice via SIRT1/HIF-1*α* pathway [16]. Gandi capsules can protect kidney function in DN mice via SIRT1/AMPK/HNF4A pathway [17]. The latest study revealed that renal injury mitigation through ferroptosis inhibition is a mechanism of action for traditional Chinese medicine. Tao-Hong-Si-Wu decoction alleviates high salt diet-induced renal inflammation and fibrosis by inhibiting ferroptosis [18]. Glabridin protects against DN by inhibiting oxidative stress and ferroptosis *in vivo* and *in vitro* [19]. Quercetin protects kidney function by inhibiting ferroptosis in renal tubular epithelial cells by regulating NRF2/HO-1 signaling pathway [20].

Jian-Pi-Gu-Shen-Hua-Yu (JPGS) decoction, consisting of *Astragalus membranaceus* Bunge, *Panax ginseng* C.A.Mey., *Rosa laevigata* Michx., *Euryale ferox* Salisb., *Cornus officinalis* Siebold & Zucc., *Dioscorea opposita* L., *Atractylodes macrocephala* Koidz., *Angelica sinensis* (Oliv.) Diels, *Salvia miltiorrhiza* Bunge, *Conioselinum anthriscoides* “Chuanxiong,” leeches, and *Rheum officinale* Baill., is commonly used in clinical practice to treat chronic kidney diseases, such as DN [21]. In this study, we established a DN mouse model to investigate the therapeutic effect of JPGS in a DN mouse model. Subsequently, we examined the effects of JPGS on ferroptosis- and GPX4 pathway-related indices. Finally, we validated whether JPGS inhibited ferroptosis in DN mice via the GPX4 pathway using GPX4 inhibitor and ferroptosis inhibitors.

## 2. Methods

### 2.1. Animals and Reagents

Specific pathogen-free grade healthy male C57BL/6J mice (6–8 weeks, 20 ± 2 g) were purchased from HFK Bioscience Co., Ltd. (Beijing, China). Detailed information on reagents, kits, and antibodies is provided in the supplemental material (available here). JPGS was acquired from Cangzhou Hospital of Integrated Traditional Chinese Medicine and Western Medicine. Quality control of JPGS was performed using ultra performance liquid chromatography coupled with mass spectrometer. Further information on JPGS can be found in the supplementary material.

### 2.2. DN Mouse Models

All experiments were conducted in an SPF environment. Each cage housed five mice with free access to food and water. This study was approved by Cangzhou Hospital of Integrated Traditional Chinese Medicine and Western Medicine of Hebei Province (approval no. CZX2023-KY-065), and all animal experiments were conducted in compliance with the “Guide for the Care and Use of Laboratory Animals” published by National Institutes of Health.

Prior to establishment of the DN mouse models, all mice underwent 1 week of adaptive feeding and then received a high-sugar high-fat diet (HFD) for 8 weeks. At the end of week 8, all mice were fasted for 12 h with unrestricted access to water and then were intraperitoneally injected with 30 mg/kg of streptozotocin (STZ). The random blood glucose levels were detected after 72 h. A blood glucose level ≥ 16.7 mmol/L was considered as the modeling criterion for type 2 diabetes mellitus (T2DM). The mice were tested weekly for 24 h urinary total protein (UTP). A blood glucoselevel ≥ 16.7 mmol/L and positive results for glucose and protein in the urine were considered as the modeling criteria for DN.

### 2.3. Animal Experiment

For the investigation of JPGS's therapeutic impact on DN mice and its potential antiferroptosis mechanism, a total of 60 mice were categorized into different groups, control, DN, irbesartan (IRB), low-dose JPGS (JPGS-L), medium-dose JPGS (JPGS-M), and high-dose JPGS (JPGS-H), each containing 10 mice. While the control group was given a regular diet, DN mouse models were established in all groups except the control. Subsequently, mice in the control and DN groups received 0.2 mL of the vehicle daily through gavage. In the IRB group, mice were administered 30 mg/kg of IRB daily via gavage. Meanwhile, the JPGS-L, JPGS-M, and JPGS-H groups received 2.4 g/kg, 4.8 g/kg, and 9.6 g/kg of JPGS daily through gavage, respectively. The duration of administration in each group was 8 consecutive weeks.

To validate whether JPGS inhibited ferroptosis in DN mice via the GPX4 pathway based on GPX4 and ferroptosis inhibitors, 60 mice were divided into groups of 10 for the control, DN, JPGS, Fer-1 (ferrostatin-1, a ferroptosis inhibitor), RSL-3 (Ras-selective lethal small molecule 3, a GPX4 inhibitor), and RSL-3+JPGS groups. The duration of administration for each group was eight consecutive weeks according to the following regimen. The mice in the control group accepted normal diets, and DN mouse model was established in all groups except for the control group. Subsequently, mice in the control and model groups received vehicle both via gavage and intraperitoneal (IP) injection. Mice in the JPGS group received 9.6 g/kg JPGS daily via gavage and vehicle via IP injection. Mice in the Fer-1 group received 10 mg/kg Fer-1 via IP injection and vehicle daily via gavage [22]. Mice in the RSL-3 group received 10 mg/kg RSL-3 via IP injection and vehicle daily via gavage [22]. Mice in the RSL-3+JPGS group received 10 mg/kg RSL-3 via IP injection and 9.6 g/kg JPGS daily via gavage.

At weeks 0, 2, 4, 6, and 8 after drug administration and fasting, the fasting blood glucose (FBG) levels and body weights of mice in each group were measured. At week 8, 24 h UTP was tested again in each group. Then, blood was collected via inner canthus, and serums were separated. After blood collection, mice were euthanized and the kidneys were removed. The left one was frozen, and the right one was immobilized in a 4% paraformaldehyde solution for later use.

### 2.4. Biochemical Experiment

Serum creatinine (Cr) and blood urea nitrogen (BUN) levels from each group were measured using corresponding assay kits.

The left kidneys were analyzed to measure the levels of malondialdehyde (MDA), ROS, iron (Fe), and the reduced glutathione (GSH) to oxidized glutathione (GSSG, glutathione disulfide) ratio (GSH/GSSG ratio) using MDA, ROS, and Fe assay kits, respectively. The remaining left kidneys were cryopreserved in an ultradeep freezer at -80°C for later use.

### 2.5. Enzyme-Linked Immunosorbent Assay (ELISA)

The levels of interleukin-1*β* (IL-1*β*), interleukin-6 (IL-6), tumor necrosis factor alpha (TNF-*α*), and 4-hydroxynonenal (4-HNE) in the kidney were measured using specific assay kits. All procedures were carried out in accordance with instructions provided by the manufacturer.

### 2.6. Morphological Observation of Pathological Changes in Kidney Tissues

The kidneys immobilized in 4% paraformaldehyde solution were sliced, sealed, and microscopically analyzed. The pathological morphology was observed using hematoxylin and eosin (HE) staining. Periodic acid Schiff (PAS) staining-positive area indicated glycogen deposition, and TUNEL (TdT-mediated dUTP nick-end labeling) staining-positive cells indicated cellular apoptosis.

### 2.7. Quantitative Reverse Transcription Polymerase Chain Reaction (RT-qPCR)

Total RNA was extracted from the kidney and reverse transcribed into cDNA. Gene expression analysis was performed using a real-time PCR detection system and the SuperReal PreMix Plus kit. Cycle thresholds (Ct) were determined, and the relative expression quantities of the genes of interest were calculated by the 2^-*ΔΔ*Ct^ method [23]. The primer sequences were included in the supplementary material.

### 2.8. Western Blot

Total protein was extracted from the kidney, and the concentration of protein was measured using bicinchoninic acid assay method. The protein was fully denatured in 99°C for 5 min. The protein samples were separated by SDS-PAGE and transferred to PVDF membranes. After blocking for 2 h in 5% skim milk, a suitable concentration of primary antibody was added and incubated overnight. After washing, the corresponding secondary antibodies were added and incubated for 2 h. Finally, membranes were washed and the bands visualized by enhanced chemiluminescence. ImageJ was utilized to quantitate the optical density of each protein band.

### 2.9. Statistics

The statistical analysis was performed using the SPSS v26.0, and all data were described in mean ± standard deviation (SD). The multigroup comparisons were performed by one-way analysis of variance. The intergroup pairwise comparisons were performed using the least significant difference test in the case of homogeneity of variance and the Tamhane's T2 test in the case of heterogeneity of variance. *P* < 0.05 indicated statistically significant differences.

## 3. Results

### 3.1. Therapeutic Effect of JPGS in DN Mice

**Figure 1:**
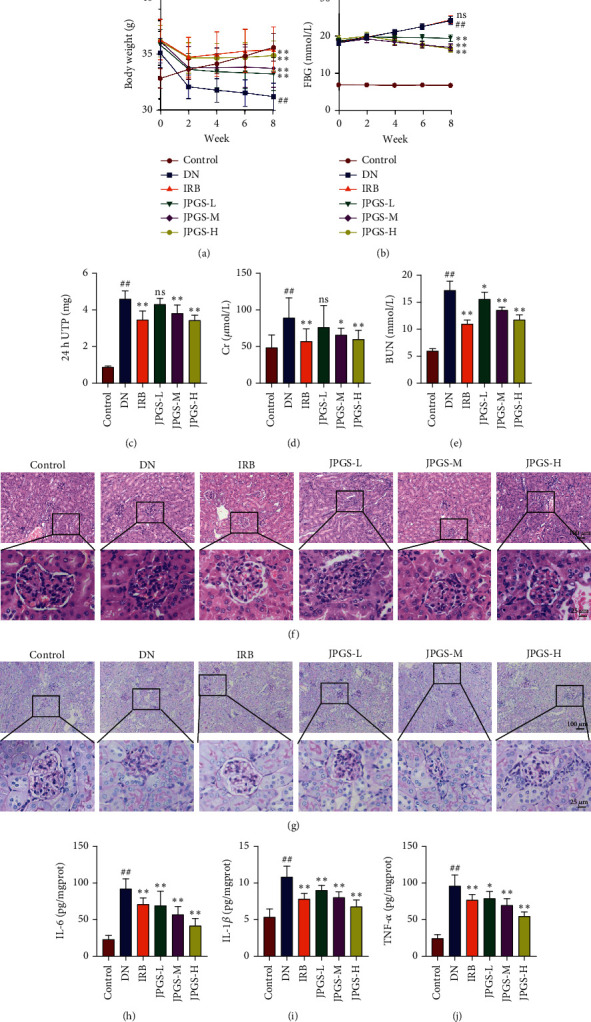
The administration of JPGS showed a therapeutic effect in mice with DN. The administration of JPGS controlled body weight, fasting blood glucose levels, and kidney function in DN mice. The changes in (a) body weight and (b) fasting blood glucose levels for each group are shown in the curve graphs. After JPGS treatment, there were improvements in kidney function, as seen in the changes in (c) 24 h UTP, (d) Cr, and (e) BUN. Kidney tissue samples stained with (f) HE and (g) PAS were also analyzed. JPGS treatment resulted in a reduction of proinflammatory cytokines (h) IL-1*β*, (i) IL-6, and (j) TNF-*α* in the kidney. Control, model, IRB, JPGS-L, JPGS-M, and JPGS-H (*n* = 10 per group) groups. Data are presented as mean ± SD. ^##^*P* < 0.01 vs. control group. ^∗^*P* < 0.05 and ^∗∗^*P* < 0.01 vs. model group.

Compared to the control group, the body weight of mice in the DN group significantly decreased. The body weight increased to varying degrees in the IRB and JPGS groups compared with the DN group (Figure 1(a)). FBG levels were elevated in the DN group compared with the control group but decreased in the IRB and JPGS groups (Figure 1(b)).

The renal function test results revealed that 24 h UTP concentration, serum Cr, and BUN levels increased in the DN group compared with the control group and decreased in the IRB, JPGS-M, and JPGS-H groups as compared to the DN group (Figures 1(c)–1(e)).

The HE staining revealed clearly visible and intact renal structures in the control group. However, severe pathological changes like glomerular mesangial cell proliferation, thickened basement membrane, tubular atrophy, and tubulointerstitial inflammatory cell infiltration were present in the DN group. The glomerular and renal tubular lesions were reduced with the most noticeable reduction in the IRB, JPGS-M, and JPGS-H groups compared with the DN group (Figure 1(f)). The results of PAS staining showed that the positive area in the control group was mainly scattered along the walls of glomerular capillaries and renal tubules, and there was no positive area in between. Excessive PAS-positive area was observed in glomerular basement membrane and mesangial area in DN mice, with the most noticeable reduction occurring in the IRB, JPGS-M, and JPGS-H groups as compared to the DN group (Figure 1(g)). Collectively, the results signified that JPGS effectively reduced pathological changes in the kidneys of DN mice.

The inflammatory factor test results revealed that IL-1*β*, IL-6, and TNF*α* concentrations increased in the DN group as compared to the control group and decreased to varying degrees in the IRB, JPGS-L, JPGS-M, and JPGS-H groups as compared to the DN group (Figures 1(h)–1(j)).

### 3.2. Effects of JPGS on Ferroptosis-Related Expression Factors in DN Mice

**Figure 2:**
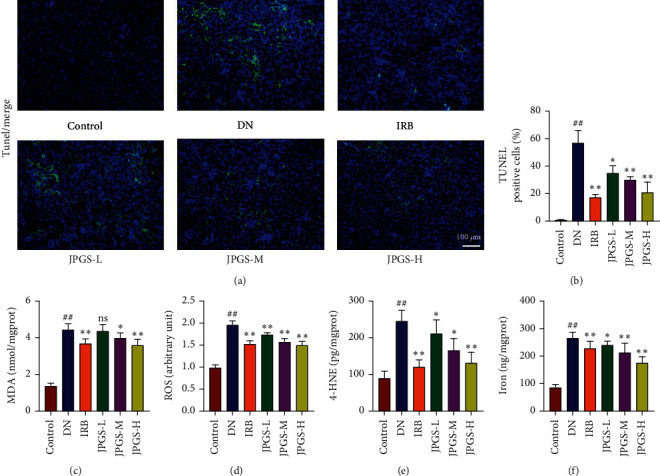
The administration of JPGS reduced renal ferroptosis of DN mice. (a, b) TUNEL staining demonstrated that JPGS treatment improved apoptosis in the kidney. The administration of JPGS decreased the levels of (c) total iron, (d) ROS, and (e, f) lipid peroxide in the kidney. Control, model, IRB, JPGS-L, JPGS-M, and JPGS-H (*n* = 10 per group) groups. Data are presented as mean ± SD. ^##^*P* < 0.01 vs. control group. ^∗^*P* < 0.05 and ^∗∗^*P* < 0.01 vs. model group.

The TUNEL staining revealed green fluorescence in the DN group as compared to the control group signaling that apoptosis had occurred in the kidneys. Positive fluorescence was markedly diminished in the IRB, JPGS-L, JPGS-M, and JPGS-H groups as compared to the DN group (Figures 2(a) and 2(b)). The oxidative stress test results revealed that ROS, MDA, and 4-HNE levels were markedly elevated in the DN group as compared to the control group. ROS, MDA, and 4-HNE levels decreased significantly in the IRB and JPGS groups as compared to the DN group (Figures 2(c)–2(e)). The changes in total iron levels were measured revealing that the Fe levels increased in the DN group as compared to the control group but decreased in the IRB, JPGS-L, JPGS-M, and JPGS-H groups as compared to the DN group (Figure 2(f)).

### 3.3. Effects of JPGS on GPX4 Pathway-Related Factors in DN Mice

**Figure 3:**
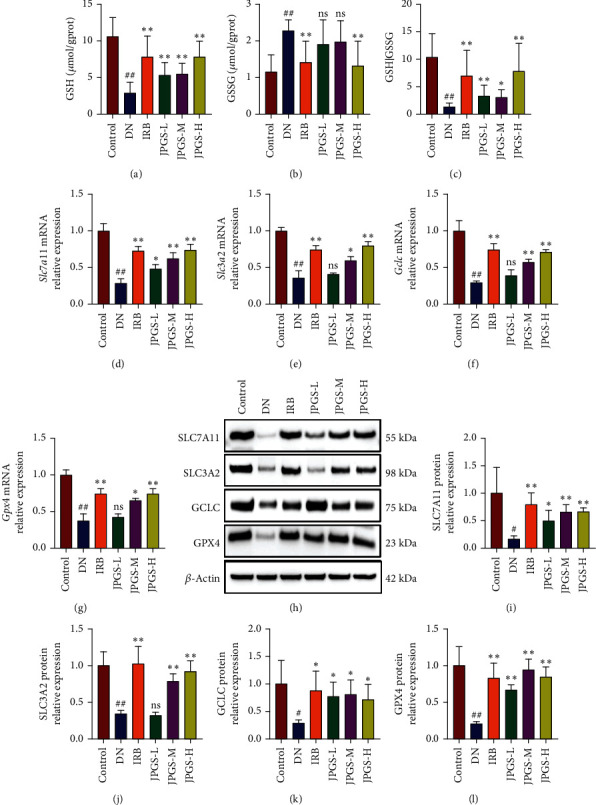
JPGS ameliorated GPX4 pathway in the kidney of DN mice. (a–c) Administration of JPGS improved the GSH/GSSG ratio in the kidney. (d–g) JPGS altered the expression levels of mRNA related to GPX4 pathway in the kidney. (h–l) JPGS altered the expression levels of proteins related to GPX4 pathway in the kidney. Control, model, IRB, JPGS-L, JPGS-M, and JPGS-H (*n* = 10 per group) groups. Data are presented as mean ± SD. ^##^*P* < 0.01 vs. control group. ^∗^*P* < 0.05 and ^∗∗^*P* < 0.01 vs. model group.

Lipid peroxidation regulated via the GPX4 pathway is vital for activating ferroptosis. The changes in GSH/GSSG ratio in the kidneys from each group were measured. The GSH/GSSG ratio decreased in the DN group as compared to the control group suggesting that the antioxidant capacity decreased and lipid peroxides accumulated in the DN mice, potentially causing ferroptosis. GSH/GSSG ratio increased in the IRB, JPGS-L, JPGS-M, and JPGS-H groups as compared to the DN group, indicating that the antioxidant capacity increased in the DN mice after drug administration (Figures 3(a)–3(c)).

The alterations in GPX4 pathway-related components were assessed using both qPCR and western blotting methods. Specifically, the solute carrier family 7A11 (SLC7A11), SLC3A2, glutamate-cysteine ligase (GCLC), GPX4 mRNA, and protein levels decreased in the DN group as compared to the control group and increased in the IRB, JPGS-L, JPGS-M, and JPGS-H groups as compared to the DN group, indicating that IRB and JPGS reduced lipid peroxide accumulation in the DN mice by activating the GPX4 pathway, and the protective effect may be associated with ferroptosis inhibition (Figure 3(d)–3(i)).

Overall, because the JPGS-H group showed the most significant effect on DN mice by activating the GPX4 pathway and reducing inflammation, oxidative stress, apoptosis, and ferroptosis, JPGS-H was selected for all subsequent experiments.

### 3.4. Therapeutic Effect of JPGS in DN Mice after Ferroptosis Inhibition

The previous results demonstrated that ferroptosis participated in DN progression and that the accumulation of lipid peroxides was reduced by activating the GPX4 pathway. To further investigate ferroptosis inhibition via the GPX4 pathway activation, the therapeutic effects of JPGS on DN mice treated with ferroptosis inhibitor Fer-1 and GPX4 inhibitor RSL3, respectively, were observed.

**Figure 4:**
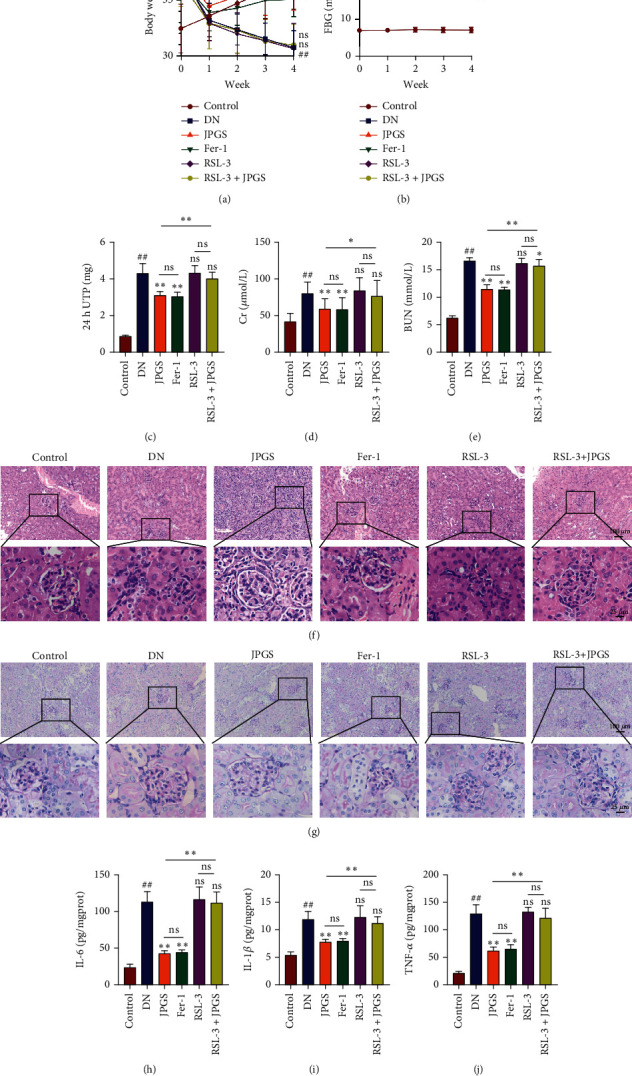
The therapeutic effect of JPGS on DN mice was abolished by GPX4 inhibitor. The administration of RSL3 completely nullified the beneficial effect of JPGS treatment on DN mice. (a, b) RSL-3 cancelled the effects of JPGS on body weight and FBG. (c–e) The levels of 24 h UTP, Cr, and BUN after treatment with JPGS. (f, g) Kidney tissue was stained with HE and PAS to evaluate histopathological changes. (h–j) RSL-3 cancelled the effect of JPSG on reducing proinflammatory cytokines in the kidney. Control, model, JPGS, Fer-1, RSL3, and JPGS+RSL3 (*n* = 10 per group) groups. Data are presented as mean ± SD. ^##^*P* < 0.01 vs. control group. ^∗^*P* < 0.05 and ^∗∗^*P* < 0.01 vs. model group.

Weekly observations were made on changes in body weight and FBG levels of all mice following the initiation of drug administration. In comparison to the control group, the DN group exhibited a decline in body weight, while the JPGS and Fer-1 groups demonstrated an increase relative to the DN group. However, no significant variation in body weights was observed between the RSL3 and RSL3+JPGS groups (Figure 4(a)). Similarly, FBG levels were higher in the DN group compared to the control group but notably reduced in the JPGS and Fer-1 groups compared to the DN group. Again, no marked distinction in FBG levels was noted between the RSL3 and RSL3+JPGS groups (Figure 4(b)).

Analysis of renal function tests indicated a rise in 24 h UTP concentration, as well as Cr and BUN levels in the DN group relative to the control group. However, these levels demonstrated a decrease in the JPGS and Fer-1 groups when compared to the DN group. Notably, no substantial disparity was observed in 24 h UTP concentration, serum Cr, or BUN levels among the DN, RSL3, or RSL3+JPGS groups (Figures 4(c)–4(e)).

The HE staining revealed that the renal structures were clearly visible and intact in the control group and that the kidneys suffered severe pathological changes like the previous study in the DN group. The renal pathological changes were relieved in the JPGS and Fer-1 groups and were not reduced in the RSL3 and RSL3+JPGS group as compared to the DN group (Figure 4(f)). PAS-positive staining was primarily distributed along the glomerular capillary wall and renal tubular wall. No PAS-positive staining was detected in the control group. PAS-positive staining was deposited in glomerular basement membrane and mesangial area in the DN group. Less PAS-positive area was observed in the glomerulus, and the thickened basement membranes were reduced in the JPGS and Fer-1 groups as compared to the DN group. The pathological changes were more severe in the RSL3 group, and no reduction was observed in the RSL3+JPGS group as compared to the DN group (Figure 4(g)).

The inflammation test results revealed that IL-1*β*, IL-6, and TNF*α* concentrations were increased significantly in the DN group as compared to the control group but decreased in the JPGS and Fer-1 groups. There was no reduction in the RSL3 and RSL3+JPGS groups as compared to the DN group (Figures 4(h)–4(j)).

### 3.5. Effects of JPGS on Ferroptosis-Related Expression Factors in DN Mice after Ferroptosis Inhibition

**Figure 5:**
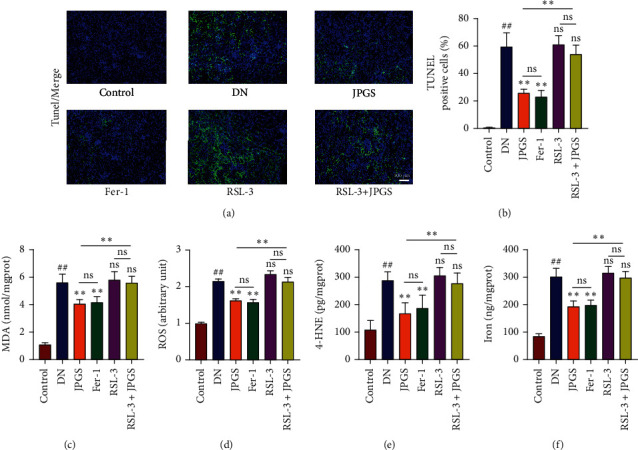
The protective effects of JPGS on ferroptosis were abolished by GPX4 inhibitor. (a, b) TUNEL staining showed that the antiapoptotic effect of JPGS treatment on DN kidney tissue was cancelled by RSL3. (c–f) The beneficial effects of JPGS treatment on total iron, ROS, MDA, and 4-HNE in the kidney were cancelled by RSL3. Control, model, JPGS, Fer-1, RSL3, and JPGS+RSL3 (*n* = 10 per group) groups. Data are presented as mean ± SD. ^##^*P* < 0.01 vs. control group. ^∗^*P* < 0.05 and ^∗∗^*P* < 0.01 vs. model group.

Positive TUNEL staining indicated by green fluorescence in the DN group as compared to the control group revealed that apoptosis had occurred. Positive fluorescence was diminished in the JPGS and Fer-1 groups. No significant difference was found in the RSL3 and RSL3+JPGS groups compared with the DN group (Figures 5(a) and 5(b)). The oxidative stress test results indicated that ROS and lipid peroxide levels increased in the DN group as compared to the control group. The levels of oxidative stress-related indicators decreased in the JPGS and Fer-1 groups as compared to the DN group. All these levels were not reduced in the RSL3 and RSL3+JPGS groups (Figures 5(c)–5(e)). Measurement of Fe level changes indicated that Fe levels were elevated in the DN group as compared to the control group and decreased in the JPGS and Fer-1 groups. No significant reduction in the RSL3 and RSL3+JPGS groups as compared to the DN group (Figure 5(f)).

### 3.6. Effects of JPGS on the GPX4 Pathway in DN Mice after Ferroptosis Inhibition

**Figure 6:**
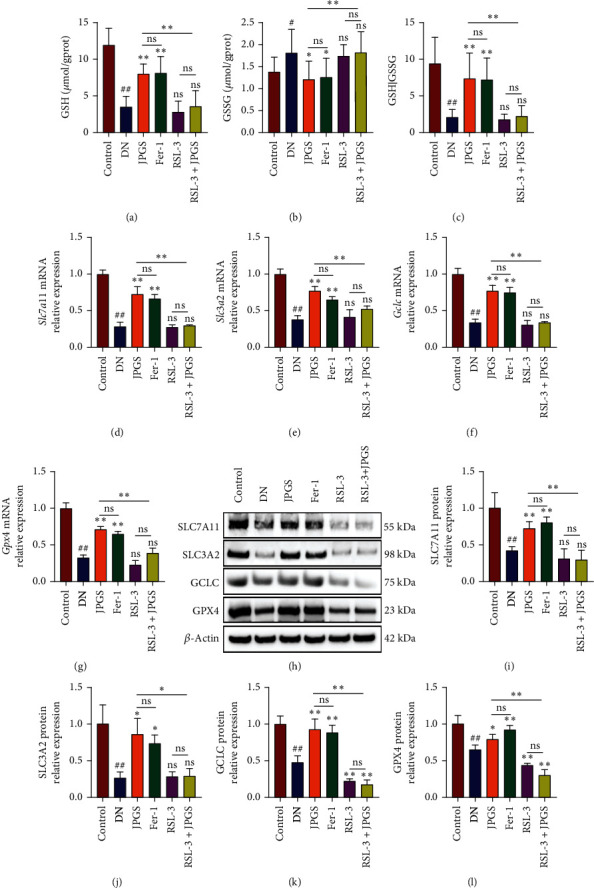
The inhibitory effect of GPX4 abolished effect of JPGS on GPX4 pathway in the kidneys. (a–c) The ameliorated GSH/GSSG ratio in DN kidney by JPGS was cancelled by RSL3 treatment. (d–g) RSL-3 cancelled the effects of JPGS on mRNA related to GPX4 pathway in DN kidney. (h–l) Western blotting revealed the expressions of GPX4 pathway-related proteins in DN kidney. Control, model, JPGS, Fer-1, RSL3, and JPGS+RSL3 (*n* = 10 per group) groups. Data are presented as mean ± SD. ^#^*P* < 0.05 and ^##^*P* < 0.01 vs. control group. ^∗^*P* < 0.05 and ^∗∗^*P* < 0.01 vs. model group.

The GSH/GSSG ratio decreased in the DN group as compared to the control group and increased in the JPGS and Fer-1 groups. No reduction was found in the RSL3 and RSL3+JPGS groups as compared to the DN group. The changes in GPX4 pathway were also evaluated, and the results demonstrated that SLC7A11, SLC3A2, GCLC, GPX4, mRNA, and protein levels decreased in the DN group as compared to the control group and increased in the JPGS and Fer-1 groups. No reduction was found in the RSL3 and RSL3+JPGS groups when compared to the DN group (Figure 6).

## 4. Discussion

STZ combined with HFD is commonly used to induce DN. HFD causes insulin resistance and pancreatic *β* cell dysfunction, whereas STZ destroys the remaining pancreatic *β* cells and exacerbates hypoinsulinism inducing overt hyperglycemia [24]. Long-term hyperglycemia damages the glomerular capillary wall causing pathological changes which results in increased permeability of the glomerular filtration membrane, the infiltration of macromolecular proteins and cell constituents in the urine, the occurrence of proteinuria, and renal tubulointerstitial fibrosis and interstitial inflammation [25]. 24 h UTP is the most sensitive indicator of early-stage DN-related renal injury [26]. Because Cr and BUN are metabolized and excreted via the kidneys, an increase in Cr and BUN levels is indicative of diminished renal function [27]. In this study, JPGS improved the body weight, blood glucose, 24 h urinary protein content, and Cr and BUN levels in DN mice. In addition, pathological staining revealed that JPGS reduced the pathological changes like glomerular mesangial matrix hyperplasia and thickened basement membrane. Long-term hyperglycemia led to the formation of an inflammatory microenvironment in the kidneys with elevated inflammatory factor levels causing further damage to kidney tissues [28]. IL-1*β*, IL-6, and TNF-*α* levels were closely associated with DN progression [29]. Furthermore, JPGS reduced IL-1*β*, IL-6, and TNF-*α* levels in the kidneys of DN mice. These results indicate that JPGS reduced disease symptoms in DN mice [30, 31].

Cell apoptosis remained fundamental to DN progression, and hyperglycemia facilitated apoptosis in various cells such as proximal renal tubular epithelial cells in DN mice [32]. The renal biopsy in patients with DN has shown that renal tubular epithelial cells, endothelial cells, and interstitial cells suffer apoptosis [33]. The protective effect against DN is demonstrated by inhibiting glucose-induced apoptosis of endothelial and capsular cells [34]. TUNEL staining is frequently used to observe cell apoptosis. In this study, TUNEL staining indicated that JPGS effectively reduced apoptosis in the kidneys of DN mice. Oxidative stress is considered a major feature of diabetic renal injury in the presence of abnormal metabolism and hemodynamics [35]. If excessive ROS is not regulated by the intrinsic antioxidant system, oxidative stress occurs. As a result, bioactive molecules (carbohydrates, proteins, lipids, and DNA) are oxidized leading to cell injuries [36]. MDA and 4-HNE, which are final products of the reaction between ROS and lipids, are highly cytotoxic, and their levels directly indicate oxidative stress levels [37]. In this study, the levels of ROS and lipid peroxide increased in the DN group as compared to the control group. JPGS effectively reduced oxidative stress and MDA, ROS, and 4-HNE levels. The above results further demonstrate that JPGS may significantly treat DN.

Iron is a metal that partakes in redox reactions in the human body. Normally, iron homeostasis is coordinated and maintained via iron metabolism involving iron absorption, utilization, recirculation, and storage, through the coordination of multiple proteins. To avoid intracellular iron accumulation and maintain intracellular iron homeostasis, normal histiocytes discharge excess iron via ferroportin, which is the only free iron exporter on the cell membrane [38]. A change in the expression of proteins involved in iron metabolism potentially induces iron metabolism disorders, rendering it impossible to discharge free intracellular iron and leading to an aberrant increase in the labile iron pool, iron overload, and the production of ROS via the Fenton reaction. The accumulation of ROS leads to GSH depletion and GPX4 inactivation. Consequently, lipids are peroxidized, and eventually, ferroptosis is induced [39, 40]. Clinical studies have shown that when cell ferroptosis occurred, SLC7A11 and GPX4 levels decreased significantly in the renal tubules of patients with DM, while the ferroptosis inhibitor effectively reduced renal tubular injuries [41]. Animal experiments in the STZ-induced DN mice model have also shown that GPX4 expression levels decreased, while lipid peroxide and iron concentrations increased [42]. Our findings suggest that JPGS reduced the total iron concentration in DN mice kidneys, signaling that iron overload was reduced.

The GPX4 pathway serves as a major defense against lipid peroxidation because GPX4 is the only known enzyme to reduce phospholipid hydroperoxides [43]. Normally, extracellular cysteine is transported into cells by system xc^−^, a cystine/glutamate antiporter consisting of SLC7A11 and SLC3A2 located on the cell membrane [44]. It is transformed into GSH by a series of enzymatic reactions including glutamate-cysteine ligase and glutathione synthetase. GSH, a reducing agent, is catalyzed by GPX4 and reduces lipid peroxides eliminating their cytotoxicity [45]. N-Acetylcysteine may activate the expression of GPX4 to reduce high glucose-induced ferroptosis in Madin-Darby canine kidney cells, and this effect may be abolished by the GPX4 inhibitor, FIN56 [46]. The results demonstrate that JPGS considerably increased GSH/GSSG ratio in DN mice kidneys, signifying that GSH depletion was reduced. Additionally, mRNA and proteins in the GPX4 pathway were elevated suggesting that JPGS promotes the expression of GPX4 in DN mice kidneys. Irbesartan, serving as a positive control, is widely utilized in the treatment of DN [47]. Our findings demonstrate that JPGS-H and irbesartan have similar effects in treating DN and in inhibiting ferroptosis. However, detailed research into irbesartan's specific action during the ferroptosis process is lacking. Studies have suggested that irbesartan may affect pathways related to oxidative stress and iron metabolism [48, 49], which aligns with the outcomes observed in our research. This lays a new groundwork for further in-depth investigation into irbesartan's role in ferroptosis-related pathways and mechanisms.

To further analyze the JPGS mechanism of action to reduce renal injury in DN, Fer-1 and RSL3 were used to verify the effects of JPGS after inhibition of GPX4 expression. Fer-1, a ferroptosis inhibitor, inhibits ferroptosis by scavenging lipid peroxides, thereby preventing their accumulation. Animal experiments showed that Fer-1 significantly reduced ROS and increased the GPX4 level in DN mice kidneys [50]. RSL3, a GPX4 inhibitor, reduced the expression and activity of GPX4 and induced ferroptosis. RSL3 has been verified to induce ferroptosis in mice [21]. The results of this study suggest that the effect of JPGS and Fer-1 alone has no significant difference in DN mice and that RSL3 abolished the therapeutic effect of JPGS, suggesting that JPGS treats DN via upregulating the expression of GPX4 pathway-related proteins, regulating iron intake, and relieving iron overload to reduce ferroptosis and improve renal injury. However, as a preliminary study, our experiment has some limitations. In future research, we plan to set up a combined treatment group with FER-1 and JPGS to more comprehensively explore the mechanism of action of JPGS on ferroptosis.

## 5. Conclusion

In summary, JPGS has significant therapeutic and anti-inflammatory effects on DN mice, and its mechanism is mainly achieved by upregulating the expression of GPX4 pathway-related proteins, thereby alleviating iron overload and ultimately reducing ferroptosis.

## Data Availability

The data used to support the findings of this study are available from the corresponding author upon reasonable request.

## Abbreviations

DN: Diabetic nephropathy
GPX4: Glutathione peroxidase 4
ROS: Reactive oxygen species
JPGS: Jian-Pi-Gu-Shen-Hua-Yu decoction
24 h UTP: 24 h urinary total protein
Cr: Creatinine
BUN: Blood urea nitrogen
MDA: Malondialdehyde
GSH: Glutathione
GSSG: Oxidized glutathione
IL-1*β*: Interleukin-1*β*
IL-6: Interleukin-6
TNF-*α*: Tumor necrosis factor alpha
4-HNE: 4-Hydroxynonenal
SLC7A11: Solute carrier family 7A11
GCLC: Glutamate-cysteine ligase.

## Supplementary Materials

Supplementary Materials The details of the materials, reagents, primers, and UPLC-MS analysis protocol can be found in the supplementary materials.
