# Supplementary material for: Jian-Pi-Gu-Shen-Hua-Yu Decoction Alleviated Diabetic Nephropathy in Mice through Reducing Ferroptosis
Source: J Diabetes Res. 2024 Mar 16;2024:9990304. doi: 10.1155/2024/9990304 (PMC10960652; doi:10.1155/2024/9990304)
Supplement: Supplementary Materials — The details of the materials, reagents, primers, and UPLC-MS analysis protocol can be found in the supplementary materials. [file 9990304.f1.docx]

**Supplementary Material**

**1 Reagents** **and assay kits**

Jian-Pi-Gu-Shen-Hua-Yu decoction (JPGS) was prepared from the pharmacy department of Cangzhou Hospital of Integrated Traditional Chinese and Western Medicine. Streptozotocin (S17049), irbesartan (S42406), and Ferrostatin-1 (S81461) were purchased from Shanghai yuanye Bio-Technology Co., Ltd. 1S,3R-RSL 3 (R873890) was purchased from Shanghai Macklin Biochemical Technology Co., Ltd. Assay kits for total protein, creatinine (Cr) and blood urea nitrogen (BUN), ROS, total iron, malondialdehyde (MDA) were purchased from Nanjing Jiancheng Biological Engineering Institute. Terminal deoxynucleotidyl transferase dUTP nick end labeling (TUNEL) staining assay kit was purchased from Beyotime Biotechnology. ELISA kits for4-hydroxynonenal, Interleukin (IL) -6, IL-1β, and tumor necrosis factor α (TNF-α) were purchased from Shanghai Enzyme-linked Biotechnology Co., Ltd. Primary antibodies for SLC7A11 (ab307601), SLC3A2 (ab303510), GPX4 (ab125066), GCLC (ab207777), and ACTB (ab6276), and secondary antibody goat anti-rabbit IgG H&L (ab205718) were purchased from Abcam.

**Supplementary Table 1 Primer sequence**

| **Genes** | **Primer sequence (5’-3’)** |
| --- | --- |
| ***Actb*** | Forward: CCCCTGAACCCTAAGGCCA |
|  | Reverse: ATGGCTACGTACATGGCTGG |
| ***Slc7a11*** | Forward: GGTCAGAAAGCCAGTTGTGG |
|  | Reverse: AGTATGCCCTTGGGGGAGAT |
| ***Slc3a2*** | Forward: CTCTCTGTTGCACGGTGACT |
|  | Reverse: TTATGCCAGCAGGGAGGTTG |
| ***Gpx4*** | Forward: CCGTCTGAGCCGCTTACTTA |
|  | Reverse: GTGACGATGCACACGAAACC |
| ***Gclc*** | Forward: CACAAGGACGTGCTCAAGTG |
|  | Reverse: GTCGGATGGTTGGGGTTTGT |

**2 UPLC-MS analysis of JPGS decoction**

JPGS was obtained from Cangzhou Hospital of Integrated Traditional Chinese Medicine and Western Medicine. Ultra performance liquid chromatography (UPLC) coupled with mass spectrometer (MS) was conducted as the quality control of JPGS.

**2.1 Sample preparation and extraction**

400 μL solution (Methanol : Water = 7:3, V/V) containing internal standard was added into 20 mg sample, and vortexed for 3 min. The sample was sonicated in an ice bath for 10 min and vortexed for 1 min, and then placed in -20 °C for 30 min. The sample was then centrifuged at 12000 rpm for 10 min (4 °C). And the sediment was removed, then centrifuged the supernatant at 12000 rpm for 3 min (4 °C). 200 μL aliquots of supernatant were transferred for LC-MS analysis.

**2.2 HPLC Conditions**

All samples were for two LC/MS methods. One alipuot was analyzed using positive ion conditions and was eluted from T3 column (Waters ACQUITY Premier HSS T3 Column 1.8 µm, 2.1 mm * 100 mm) using 0.1 % formic acid in water as solvent A and 0.1 % formic acid in acetonitrile as solvent B in the following gradient: 5 to 20 % in 2 min, increased to 60 % in the following 3 mins, increased to 99 % in 1 min and held for 1.5 min, then come back to 5 % mobile phase B witnin 0.1 min, held for 2.4 min. The analytical conditions were as follows, column temperature, 40 °C; flow rate, 0.4 mL/min; injection volume, 4 μL; Another alipuot was using negative ion conditions and was the same as the elution gradient of positive mode.

**2.3 MS Conditions (AB)**

The data acquisition was operated using the information-dependent acquisition (IDA) mode using Analyst TF 1.7.1 Software (Sciex, Concord, ON, Canada). The source parameters were set as follows: ion source gas 1 (GAS1),50 psi; ion source gas 2 (GAS2), 50 psi; curtain gas (CUR), 25 psi; temperature (TEM), 550 °C; declustering potential (DP), 60 V, or−60 V in positive or negative modes, respectively; and ion spray voltagefloating (ISVF), 5000 V or−4000 V in positive or negative modes, respectively. The TOF MS scan parameters were set as follows: mass range, 50–1000 Da; accumulation time, 200 ms; and dynamic background subtract, on. The product ion scan parameters were set as follows:mass range, 25–1000 Da; accumulation time, 40 ms; collision energy, 30 or−30 V in positive or negative modes, respectively; collision energy spread, 15; resolution, UNIT; charge state, 1 to 1; intensity, 100 cps; exclude isotopes within 4 Da; mass tolerance, 50 ppm; maximum number of candidate ions to monitor per cycle, 18.

**2.4 UPLC-MS analysis results of JPGS decoction**

The JPGS test solution was firstly analyzed by the UPLC-MS system. **Supplementary Figure 1** manifested the total ion chromatogram of JPGS in (A) positive ion mode and (B) negative ion mode. 28 kinds of components were identified (**Supplementary Table 2**). We found that Astragaloside IV, Ginsenoside Ro, Ginsenoside RG1, Loganin, Dioscin, Gallic acid, Oleanolic acid, Atractylenolide III, Ferulic acid, Rosmarinic acid, Salvianolic acid A, Periandrin III, Emodin, Schisandrin, and others widely existed in JPGS.

A


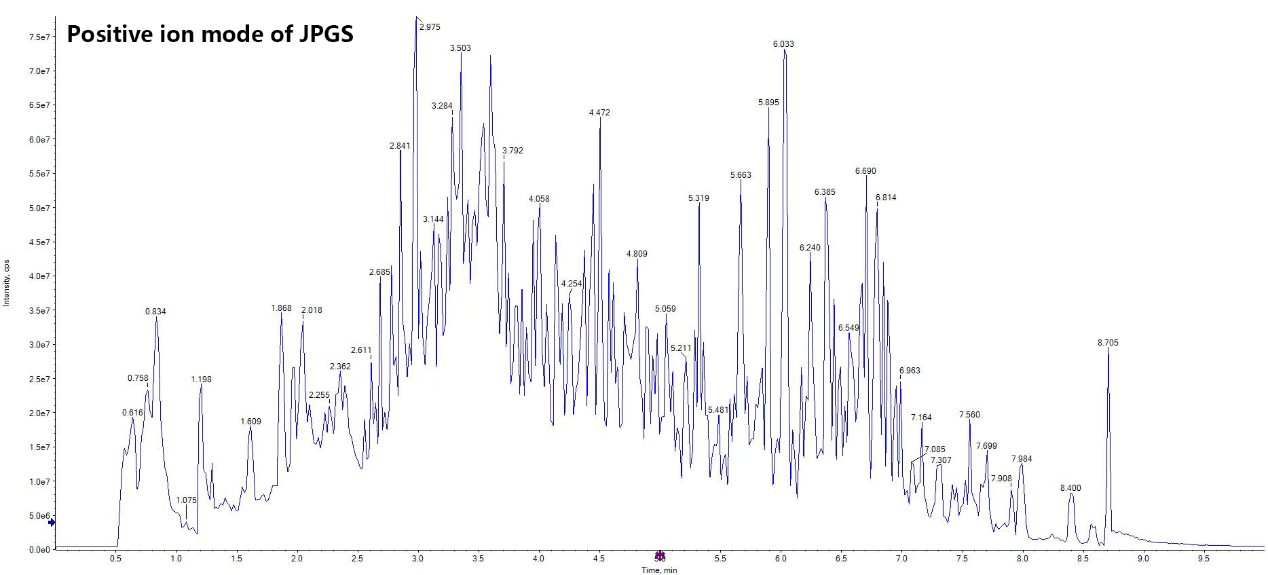


B


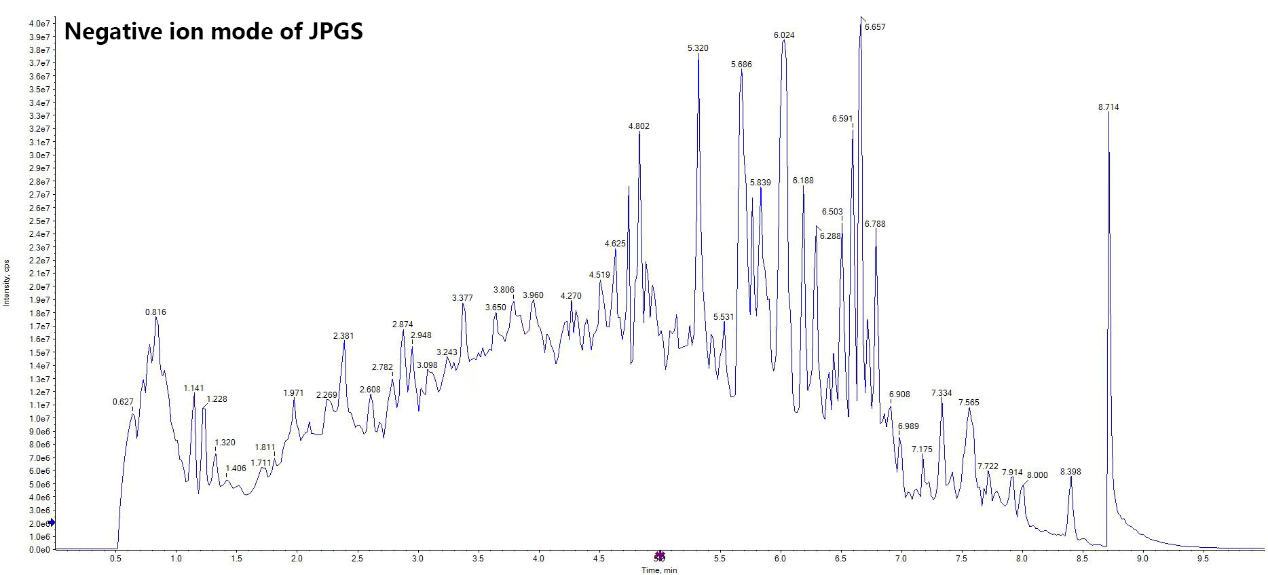


**Supplementary Figure 1** Total ion chromatogram of JPGS obtained by UPLC-MS analysis in (A) positive ion mode and (B) negative ion mode.

**Supplementary Table 2 Chromatography (TIC) of JPGS based on UPLC-MS/MS.**

| **Compounds** | **Formula** | **Mode** | **Molecular**  **weight (Da)** | **RT (min)** | **Adduct** | **score** |
| --- | --- | --- | --- | --- | --- | --- |
| Gallic acid | C7H6O5 | Negative | 170.0215 | 1.8141 | [M-H]- | 0.9315 |
| Salvianolic acid A | C26H22O10 | Negative | 494.1213 | 1.8201 | [M-H]- | 0.6864 |
| gomisin N | C23H28O6 | Negative | 400.1886 | 2.3266 | [M-H]- | 0.7215 |
| 3,6,8-Trimethylallantoin | C7H12N4O3 | Positive | 200.0909 | 2.6016 | [M+HCOO+2H]+ | 0.7489 |
| Rubschisandrin | C23H28O6 | Negative | 400.1886 | 2.8327 | [M-H]- | 0.5973 |
| Loganin | C17H26O10 | Negative | 390.1526 | 3.0686 | [M+CH3COO]- | 0.7735 |
| schidigera-saponin C1 | C44H70O18 | Positive | 886.4562 | 3.7220 | [M+]+ | 0.5559 |
| Ferulic acid | C10H10O4 | Negative | 194.0579 | 3.9605 | [M-H]- | 0.9787 |
| Isoastragaloside I | C45H72O16 | Positive | 868.482 | 4.5889 | [M+K]+ | 0.6496 |
| Astragaloside VI | C47H78O19 | Positive | 946.5137 | 4.6429 | [M+H]+ | 0.7827 |
| Astragaloside VII | C47H78O19 | Positive | 946.5137 | 5.0987 | [M+H]+ | 0.7222 |
| Astragaloside V | C47H78O19 | Positive | 946.5137 | 5.0994 | [M+Na]+ | 0.6629 |
| Ginsenoside F1 | C36H62O9 | Negative | 638.4394 | 5.2038 | [M+HCOO]- | 0.7593 |
| Astragaloside III | C41H68O14 | Positive | 784.4609 | 5.2266 | [M+Na]+ | 0.9228 |
| Astragaloside IV | C41H68O14 | Negative | 784.4609 | 5.3487 | [M-H]- | 0.9445 |
| Schisandrin | C24H32O7 | Positive | 432.2148 | 5.4747 | [M+Na]+ | 0.885 |
| Ginsenoside Rh6 | C36H62O11 | Positive | 670.4292 | 5.4780 | [M+H-H2O]+ | 0.7564 |
| Astragaloside II | C43H70O15 | Positive | 826.4715 | 5.5879 | [M+H-H2O]+ | 0.8956 |
| Oleanolic acid | C30H48O3 | Positive | 456.3603 | 5.6222 | [M+H-H2O]+ | 0.661 |
| Ginsenoside RG1 | C42H72O14 | Negative | 800.4922 | 5.8487 | [M+CH3COO]- | 0.5686 |
| Ginsenoside Ro | C48H76O19 | Positive | 956.4981 | 5.8752 | [M+NH4]+ | 0.7377 |
| Rosmarinic acid | C18H16O8 | Positive | 360.0845 | 5.9383 | [M+H-2H2O]+ | 0.6623 |
| Wuweizisu C | C22H24O6 | Positive | 384.1573 | 6.1950 | [M+H]+ | 0.7922 |
| Dioscin | C45H72O16 | Negative | 868.482 | 6.2685 | [M-H]- | 0.9166 |
| Atractylenolide III | C15H20O3 | Negative | 248.1412 | 6.2797 | [M-H]- | 0.7243 |
| Emodin | C15H10O5 | Negative | 270.0528 | 6.6038 | [2M-H]- | 0.9306 |
| Periandrin III | C42H64O16 | Positive | 824.4194 | 7.1249 | [M+]+ | 0.5421 |
| Diosgenin | C27H42O3 | Positive | 414.3134 | 7.3879 | [M+]+ | 0.7234 |
